# Dissipation, Bioconcentration and Dietary Risk Assessment of Thiamethoxam and Its Metabolites in *Agaricus bisporus* and Substrates under Different Application Methods

**DOI:** 10.3390/toxics11060500

**Published:** 2023-06-01

**Authors:** Shanshan Chen, Qicai Zhang, Qinxiong Rao, Xianli Wang, Penghui Du, Weiguo Song

**Affiliations:** 1 Institute of Agro-Food Standards and Testing Technology, Shanghai Academy of Agricultural Sciences, Shanghai 201403, China; shanshanchen2013@saas.sh.cn (S.C.); qicaizhang@126.com (Q.Z.); qinxiongrao@163.com (Q.R.); wangxianli@saas.sh.cn (X.W.); 2 College of Food Sciences, Shanghai Ocean University, Shanghai 201306, China; 18839481491@163.com

**Keywords:** *Agaricus bisporus*, thiamethoxam, clothianidin, thiamethoxam-urea, dissipation, metabolism, bioconcentration, dietary risk assessment

## Abstract

In order to acquire scientific evidence for the application of thiamethoxam (TMX) in *Agaricus bisporus* cultivation, residue and dissipation experiments for field trials were performed with the application of TMX in compost and casing soil, respectively. An effective QuEChERS method was established to analyze TMX and its two metabolites, clothianidin (CLO) and thiamethoxam-urea (TMX-urea), in compost, casing soil, and fruiting bodies. The results indicated that the TMX dissipation half-lives (t*_1_*_/*2*_) at dosages of 10 and 50 mg kg^−1^ were 19.74 d (day) and 28.87 d in compost and 33.54 d and 42.59 d in casing soil, individually. TMX, CLO, and TMX-urea were observed after TMX application in compost and casing soil. For TMX applied to the casing soil, only TMX residues were detected in fruiting bodies with bioconcentration factors (BCFs) of 0.0003~0.0009. In addition, both the chronic risk quotient (RQ) and acute risk quotient (HQ) values of TMX in fruiting bodies were far less than 1, which means the dietary health risks to humans were acceptable. However, in the TMX application to the compost, these analytes were not detected in the fruiting bodies. This suggested that the application of TMX in compost was safer than in casing soil during *A. bisporus* cultivation.

## 1. Introduction

*Agaricus bisporus* is one of the cultivated mushrooms favored by consumers all over the world. The estimated average annual output in China for the three years from 2019 to 2021 was 2 million tons, meaning that China is ranked first in the world for *A. bisporus* production as well as being the largest exporting country [1]. *A. bisporus* is highly nutritious and has several pharmaceutical functions, such as reducing oxidative damage, strengthening the immune system, suppressing the growth of cancer cells, and providing beneficial effects on cardiovascular and diabetic diseases [2,3]. However, the threat of insect pests and diseases is inevitable during the cultivation of *A. bisporus*. The pests compete with mycelium in substrates or directly damage the fruiting body, leading to yield loss [4,5]. Sciarids can reduce the *A. bisporus* yield by 15.5–21.8 kg m^−2^ [6] and have become the most important insect to control during mushroom cultivation. Therefore, the role of insecticides is indispensable as an important component of the comprehensive control strategy, although very few pesticides are registered for mushroom cultivation in many countries [7,8].

Thiamethoxam is a second-generation neonicotinoid insecticide that is widely used for sciarids control. It was registered in China in 2001 and can control a broad spectrum of pest insects, such as Diptera, Thysanoptera, Coleoptera, Hemiptera, and Lepidoptera. Thus, TMX is a potential insecticide for the chemical control of sciarids in *A. bisporus*. However, the worldwide use of TMX has received much attention with regard to the safety of its metabolites and residues. To date, many studies have revealed that TMX also has adverse impacts on non-target species such as aquatic invertebrates, birds, and honeybees [9,10,11,12]. It may induce neurobehavioral alterations and pose potential health risks to humans. [13]. The major metabolites of TMX were clothianidin (CLO), thiamethoxam-urea (TMX-urea), thiamethoxam-dm-urea (TMX-dm-urea), clothianidin-urea (CLO-urea), and clothianidin-dm-urea (CLO-dm-urea) [14]. CLO is also a neonicotinoid insecticide that is actually more toxic than TMX because of its higher lipophilicity [15]. As a consequence, imidacloprid (IMI), TMX, and CLO were banned for use on field crops outdoors in Europe [16]. In view of the global production and utilization of thiamethoxam and its low toxicity and broad-spectrum insecticidal activity, it is more suitable to be applied in relatively closed factory environments such as greenhouses and mushroom cultivations in the future.

Before that, the safety of TMX application in *A. bisporus* cultivation should be assessed. The dissipative and metabolic behaviors of TMX are the key points used to evaluate its safe application. Previous studies related to the dissipation and degradation of TMX focused on residues of the parent compound TMX in fruits and vegetables, field crops, and soils [17,18,19]. The FAO/WHO joint meeting on pesticide residues (JMPR) also identified the sum of TMX and CLO as an estimated dietary intake of plants [20]. However, only a few researchers paid attention to residues of TMX and its metabolite CLO in plants [21,22]. Currently, HPLC (high-performance liquid chromatography) and UPLC-MS/MS (ultra-performance liquid chromatography-tandem mass spectrometry) are effective detection methods for the analysis of TMX and some of its metabolites [23,24]. Until now, the synchronous detection of residues of TMX and its main metabolites CLO and TMX-urea in compost, casing soil, and the fruiting bodies of *A. bisporus* had not been conducted. Thus, a method for extraction and cleanup utilizing UPLC-MS/MS should be developed. Moreover, the behavior of pesticides would be different if the pesticide was applied in the upper-layer casing soil or lower-layer compost [25]. Environmental conditions and soil properties are considered to be the two main factors that influence TMX dissipation in soil. The soil type could influence the half-life of TMX, which ranges from 80 to 170 d [17]. It is possible that the bioconcentration and dissipation of TMX metabolites and residues in *A. bisporus* and substrates under different application methods are attributable to the distinct composition and physicochemical properties of casing soil and compost (Table S1). Consequently, the dissipation behavior of TMX and its main metabolites, CLO, and TMX-urea, and the exposure risks of TMX due to the intake of *A. bisporus* fruiting bodies under two application methods should be made clear to assess its safety and clarify the appropriate application method based on field residue tests.

To systematically explore how TMX dissipates and metabolizes in mushrooms and different substrates, and to provide a basic guideline for the safe and reasonable application of TMX, field trials were designed in an industrial *A. bisporus* factory. The purposes included: (1) creating a QuEChERS (quick, easy, cheap, effective, rugged, safe) method for determination on TMX and its main metabolites CLO and TMX-urea in *A. bisporus* ecosystem (including compost, casing soil, and fruiting body) using UPLC-MS/MS simultaneously; (2) elucidating the dissipation, metabolism, and bioaccumulation characteristics of TMX in compost or casing soil-fruiting body systems; and (3) comparing the dietary risks of TMX in *A. bisporus* fruiting bodies when TMX is administered to compost or casing soil with the two different application methods.

## 2. Materials and Methods

### 2.1. Chemicals and Reagents

The standards for thiamethoxam (99.6%), clothianidin (99.7%), and thiamethoxam-urea (99.3%) were provided by Dr. Ehrenstorfer GmbH (Augsburg, Germany). The commercial formulation of thiamethoxam water-dispersible granules (25% WDG) was purchased from Syngenta Crop Protection Co., Ltd. (Basel, Switzerland). LC-MS (liquid chromatography-mass spectrometry)-grade methanol and acetonitrile were supplied by Merck (Darmstadt, Germany). LC-MS-grade ammonium acetate was obtained from ThermoFisher Scientific (Waltham, MA, USA). The QuEChERS extraction salt packets (EN method, European Norm method) and d-SPE (dispersive solid-phase extraction) sorbent purification packets were purchased from Agilent (Agilent Technologies, Lake Forest, CA, USA).

High-level standard solutions (1000 mg L^−1^) of TMX, CLO, and TMX-urea were prepared independently in methanol. The mixed standard solutions at the required concentration were diluted with acetonitrile. All solutions were stored at −20 °C in the dark before use to avoid degradation. The multiple-level working solutions of TMX, CLO, and TMX-urea that were used to create a calibration curve were prepared with acetonitrile by gradually diluting the mixed standard solutions.

### 2.2. Field Trials with TMX Application during A. bisporus Cultivation

#### 2.2.1. *A. bisporus* Cultivation

The field trials were carried out at 121.16° E, 30.89° N in the Jinshan district of Shanghai, China, at Shanghai Lian Zhong edible fungi cooperatives. A diagrammatic representation of the growing stages and a brief description related to *A. bisporus* production are given in Figure S1. Details of the cultivation can be found in Section S1.1 of the Supplementary Materials.

#### 2.2.2. TMX Application and Sampling

Thiamethoxam (25% WDG) was applied separately in the upper-layer casing soil and lower-layer compost to monitor the metabolites and degradation rate of TMX application in compost and casing soil. The final residue trial of fruiting bodies was designed to study the bioconcentrations of TMX and its metabolites in *A. bisporus* and to perform a dietary risk assessment. All field trials were conducted in accordance with the Chinese industrial standard “Guidelines on Pesticide Residue Trials” (NY/T 788-2018). According to the recommended dosage range of 25% TMX WDG application in seed treatment (0.25–1.25 g a.i. kg^−1^) [26], with approximately 1 kg of the strain mixed with 25 kg of compost (wet weight), TMX was exposed to compost or casing soil at a possible minimum effective dosage (10 mg a.i. kg^−1^) and the maximum recommended dosage (50 mg a.i. kg^−1^). The details of TMX application and sampling in the two different methods are described in Section S1.2 of the Supplementary Materials.

### 2.3. Sample Preparation and Purification

#### 2.3.1. Compost and Casing Soil Samples

Sample preparations and purifications were completed by QuEChERS citric acid buffer (EN method) with minor modifications. Casing soil or compost dry samples weighing 5.0 g were added into 50 mL centrifuge tubes, and for spiked samples, they were fortified with mixed standard solutions at appropriate concentrations and maintained for 30 min. After 5 mL of ultrapure water was added, the centrifuge tube was shaken for a few seconds to hydrate the sample. After 10 mL of acetonitrile and the QuEChERS citric acid extraction salt packet (anhydrous MgSO_4_, 4.0 g; NaCl, 1.0 g; sodium citrate, 1.0 g; sodium hydrogen citrate, 0.5 g) were added, the mixtures were immediately vortexed vigorously for 1 min and extracted ultrasonically for 20 min, following 10 min of centrifugation at 4000 rpm. Afterwards, 6 mL of supernatant was transferred into the 15 mL centrifuge tube containing 900 mg of anhydrous MgSO_4_, 150 mg of PSA, 150 mg of C18, and 15 mg of GCB, vortexed, and cleanup with the adsorbents was performed for 1 min. Subsequently, this mixture was centrifuged at 4000 rpm for 10 min again. A total of 1 mL of supernatant was filtered through a 0.22 μm nylon membrane for UPLC-MS/MS analysis. However, some early sampling samples with high content were diluted 10–50 times according to the estimated concentration to fit the standard curve range. The contents of TMX and its metabolites calculated in all of the casing soil and compost samples were finally multiplied by the dilution factor and, based on wet weight, converted according to moisture contents (Table S1).

#### 2.3.2. Fruiting Body Samples

A 10.0 g homogenized fruiting body sample was added to a 50 mL centrifuge tube and mixed with 10 mL of acetonitrile and the QuEChERS citric acid extraction salt packet. The mixture was immediately vortexed vigorously for 1 min, and then centrifuged at 4000 rpm for 10 min. Subsequently, 6 mL of the upper-layer solution was transferred into the 15 mL centrifuge tube preloaded with the sorbents with 900 mg of anhydrous MgSO_4_, and 150 mg PSA was performed for cleanup of the interference in fruiting bodies. The mixtures were vortexed intensely for 1 min and centrifuged at 4000 rpm for 10 min again. At last, 1 mL of the supernatant was filtered through a 0.22 μm nylon membrane into an autosampler vial for UPLC-MS/MS analysis.

### 2.4. UPLC-MS/MS Analysis

**Figure 1:**
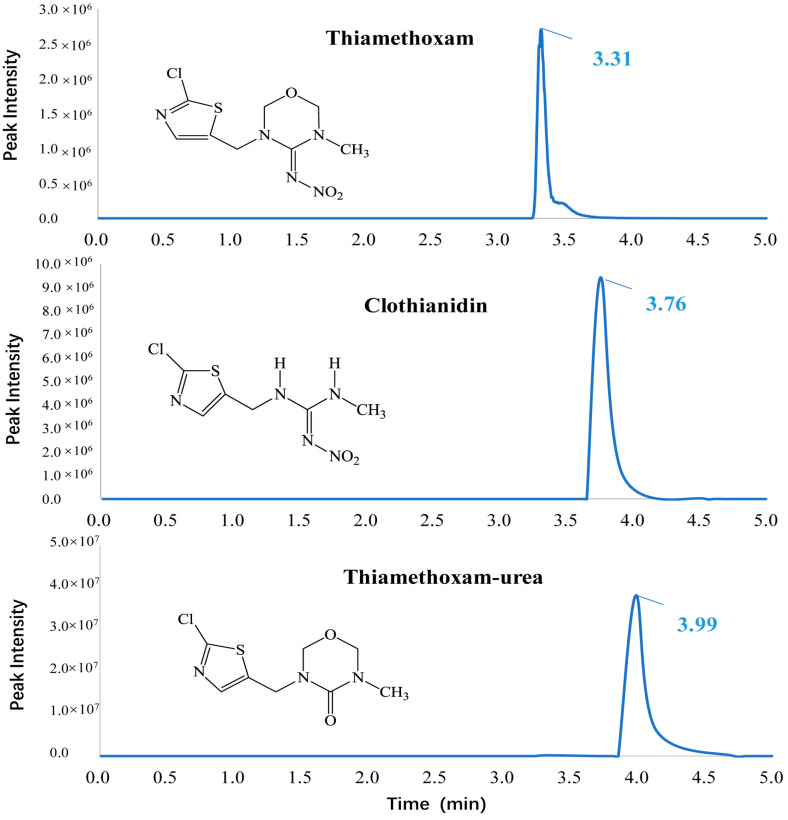
Chemical structures and UPLC-MS/MS chromatogram of thiamethoxam and its metabolites.

Thiamethoxam and its metabolites were determined on an Acquity UPLC (Waters) system connected to an AB SCIEX 5500 triple-quadrupole mass spectrometer (Framingham, MA, USA). The target analytes were separated on a C18 column (100 mm × 2.1 mm, 1.7 μm particle size) maintained at a temperature of 30 °C. The binary solvent system consisted of methanol (A) and 5 mM ammonium acetate in ultrapure water (B), and the flow rate was 0.30 mL min^−1^. Elution of the gradient was performed under the following conditions: 10% A was held for 1.0 min, changed to 60% A (in 1.0–3.5 min) and maintained for 3.5–4 min, and decreased to 10% A (4–5 min). The overall analysis time was 5 min. The injection volume was set at 3 μL. The retention times of TMX, CLO, and TMX-urea were 3.31 min, 3.76 min, and 3.99 min, respectively (Figure 1). Individual standard solutions of target analytes (1 mg L^−1^) were directly infused into the mass spectrometer to optimize the precursor ion, product ion, and collision energy (CE). Triple quadrupole MS/MS was conducted in the positive ESI mode for TMX, CLO, and TMX-urea using MRM with two mass transitions where both of the target analytes yielded [M + H]^+^ precursor ions. The high-intensity mass transition was employed for quantitation, while another mass transition was used for confirmation. The optimal MS/MS parameters of TMX, CLO and TMX-urea are shown in Table S2. The ion spray voltage was set at 4.5 kV, nitrogen was used as the nebulizer/desolvation gas, and argon was used as the collision gas. The temperature of the block source (TEM) was maintained at 500 °C, while the nebulizer gas (GS 1) and turbo gas (GS 2) pressures were set at 50 psi. The curtain gas (CUR) pressure was 35 psi, and the collision gas (CAD) value was set to 8. The AB Sciex, v1.6 analyst software was utilized for controlling instruments, data acquisition, and processing.

### 2.5. Dissipation Dynamics Behavior

The half-life period and dissipation kinetics of TMX in compost and casing soil can be expressed by first-order dynamic Equations (1) and (2) [21]:

C*_t_* = C_0_ e*^−kt^* (1)

t*_1_*_/*2*_ = ln 2/k (2)

where C_0_ represents the initial concentration of TMX, C*_t_* is the TMX concentration at the time point t (day), k is the degradation rate constant, and t*_1_*_/*2*_ represents the half-life (day).

### 2.6. Bioconcentration Factors (BCFs)

The formula for the bioconcentration factors (BCFs) of TMX and its metabolites can be expressed as follows [27]:

BCF = C*_g_* (t*_h_*)/C*_w_* (t*_h_*) (3)

where C*_g_* is the residue levels of TMX and its metabolites in a *A. bisporus* fruiting body, C*_w_* is their concentrations in compost or casing soil, and t*_h_* (day) is the time at which the samples were collected [28].

### 2.7. Dietary Risk AssessmentFigur

The final residue level in fruiting bodies at harvesting was related to a dietary risk assessment of *A. bisporus*. The chronic exposure risk assessment was performed based on the national estimated daily intake (NEDI, mg kg^−1^* bw* day^−1^), using Equation (4) as follows [21]:

NEDI = Σ (STMR*_i_* × *F_i_*)/*b_w_* (4)

where STMRi (mg kg^−1^) is the median value of the TMX residue test in *A. bisporus* fruiting bodies or the established maximum residue limit (MRL) in a certain kind of food registered in China, complying with “National food safety standard maximum residue limits of pesticides in foods”(GB 2763-2021); *Fi* (kg day^−1^) is a class of food consumption data; *bw* is the average body weight for a Chinese adult (63 kg); ADI (mg kg^−1^ *bw* day^−1^) is the acceptable daily intake of TMX.

RQ = NEDI/ADI(5)

RQ is the chronic risk quotient. Typically, RQ > 1 implies that the chronic dietary intake risk is unacceptable to the general population. RQ < 1 indicates an acceptable risk. The smaller the RQ value, the lower the risk.

On the other hand, the acute dietary risk assessment was associated with MRLs expected to be in food and short-term toxicological effects, i.e., the acute reference dose (ARfD, mg kg^−1^ *bw* day^−1^). The national estimated short-term intake (NESTI, mg kg^−1^ *bw* day^−1^) was divided by ARfD to obtain the acute risk quotient (HQ). The formula is as follows [24]:

NESTI = HR × F/*bw* (6)

HQ = NESTI/ARfD (7)

where HR (mg kg^−1^) is the highest residue level of TMX detected, and *F* (kg day^−1^) is the edible fungus consumption data for the general Chinese population. When HQ > 1, it means that the acute dietary risk is unacceptable. When HQ < 1, it represents an acceptable acute risk to the national general population.

## 3. Results and Discussion

### 3.1. Method Validation

#### 3.1.1. Linearity, Matrix effect (ME), Limit of Detection (LOD), and Limit of Quantification (LOQ)

Ideal correlation coefficients (r > 0.99) and linear calibration curves were observed over the concentration range of TMX and CLO in casing soil and compost of 0.005~1 mg L^−1^, while in fruiting bodies they were observed at concentrations of 0.001~1 mg L^−1^. Furthermore, the concentration range of TMX-urea in casing soil and compost was 0.001~0.5 mg L^−1^, while in fruiting bodies it was 0.0005~0.5 mg L^−1^. Related results are presented in Table 1. We diluted the sample out of the linear range to the appropriate concentration to match the standard curve range. Matrix effects (ME) in all three matrices were usually achieved by the slope ratios of the target analyte calibration curve equation in the relevant matrices and in the solvent. ME was calculated by means of Equation (8):

ME (%) = (slope*_matrix_*/slope*_solvent_* − 1) × 100% (8)

Most of the target analytes showed matrix enhancement effects (3.0~23.2%), except for CLO and TMX-urea in compost, which demonstrated matrix suppression effects (−14.9% and −22.2%, respectively) (Table 1). In general, the improvement or suppression results, were due to the incomplete removal of pigments, polysaccharides, lipids, fatty acids and other interfering substances [29]. The most commonly used method to compensate for matrix effect errors in analysis is to utilize matrix-matched calibration standards, and the results proved that they are indispensable for accurate quantification [30]. Therefore, the blank matrix solutions processed according to Section 2.3 were used to prepare matrix-matched standard curves.

In this study, we defined the LODs as the lowest concentration levels of the matrix-matched standard curves in three matrices. Moreover, the LOQs of TMX, CLO, and TMX-urea were determined based on the minimum validation spiked levels, as seen in Table 1.

#### 3.1.2. Accuracy and Precision by Recovery Experiments

The accuracy and precision of the established method were investigated via recovery experiments. The real concentration of TMX and its metabolites determined by means of the complete pretreatment was compared with those initially fortified to the three blank matrices at five levels, 5, 1, 0.1, and 0.02 mg kg^−1^, and LOQs for six replicates (*n* = 6). As shown in Table S3, the average recoveries of TMX in three matrices ranged from 88.6% to 113.4% with RSDs of 3.5~12.1%. The average recoveries ranged from 86.1 to 103.5% with RSDs of 3.1~13.7% for CLO, and 83.8 to 101.2% with RSDs of 2.8~14.1% for TMX-urea, respectively. The results were considered satisfactory according to the guidelines for the national standards of pesticide residue detection methods [31].

### 3.2. Dissipation Dynamics of TMX in Compost and Casing Soil

**Figure 2:**
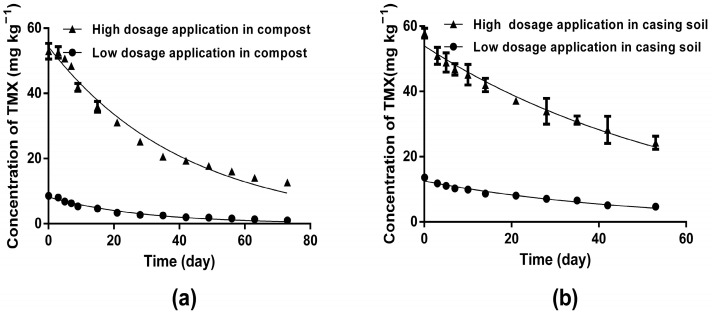
The dissipation dynamic of thiamethoxam (TMX) in *A. bisporus* cultivation. (**a**) The dissipation dynamic of TMX in compost at two dosage groups. (**b**) The dissipation dynamic of TMX in casing soil at two dosage groups. Each treatment was performed in three replicate plots; data displayed are mean ± SD (*n* = 3).

Studies on dissipation dynamics play a significant role in dietary risk assessment and were conducive to evaluating the safe and reasonable application of TMX in *A. bisporus* cultivation. Dissipation curves for TMX in compost and casing soil applied at two dosages were plotted using the model of first-order kinetics as presented in Table 2 and Figure 2. The initial residue contents of TMX in compost were 8.576 mg kg^−1^ and 52.92 mg kg^−1^ in the low- and high-dosage groups, respectively. At the end of the field trial, the final residue contents decreased to 1.023 mg kg^−1^ and 12.661 mg kg^−1^, respectively. On the other hand, the initial contents of TMX residues in casing soil were 13.633 mg kg^−1^ and 57.886 mg kg^−1^ in the low- and high-dosage groups, respectively; at 53 d after TMX was applied in casing soil, the final residues decreased to 4.673 mg kg^−1^ and 24.252 mg kg^−1^, respectively. This means that the final degradation rates of TMX in compost were 88.1% and 76.1% at dosages of 10 mg kg^−1^ and 50 mg kg^−1^, respectively, while the degradation rates were only 65.7% and 58.1%, respectively, in casing soil. According to the kinetic equations shown in Table 2, the estimated half-lives (t*_1_*_/*2*_) of TMX were 19.74 d and 28.87 d at dosages of 10 mg kg^−1^ and 50 mg kg^−1^ in compost, respectively, yet they were 33.54 d and 42.89 d in casing soil, respectively. It is worth noting that the half-life in casing soil was longer than in compost, which might be attributed to *A. bisporus* compost containing much more organic material (65.9 ± 1.6%) that is conducive to the growth of microorganisms. It was reported that the dissipation rate of TMX depended on a variety of factors, including soil characteristics, pH values, application dose, climatic or cultivation conditions, microorganisms, organic fertilizer use, and so on [32,33]. The half-lives of TMX vary from 3.9 d to 94.1 d, or even longer periods, in different soil types and plant ecosystems such as pepper, citrus, tobacco, and Swiss chard [22,34,35]. The persistence of TMX in soil can be affected by introducing organic amendments such as biochar and microbial activity to accelerate degradation by *Pseudomonas* and *Bacillus* [36,37]. Inorganic biostimulators also had a different impact on the rate of pesticide degradation [38]. Piotr Iwaniuk reported that titanium/silicon shortened the dissipation time of four fungicides in wheat plants. Titanium resulted in the greatest reduction in dissipation time of spiroxamine by 70%, followed by triadimenol (43%) and tebuconazole (37%). Silicon shortened the degradation of spiroxamine (69%) and tebuconazole (52%) the most. This might have occurred in our study due to the high content of organic materials and the low pH value in the compost, which was more favorable to the reproduction of microorganisms than the casing soil, thus accelerating the degradation of TMX and shortening the half-life.

### 3.3. Residual Fate of CLO and TMX-Urea in Compost and Casing Soil

**Figure 3:**
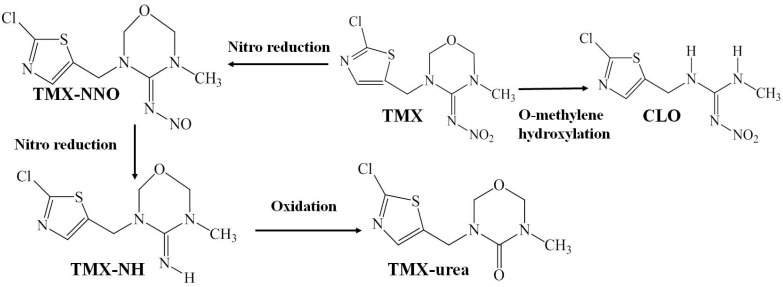
Potential metabolic pathways for thiamethoxam (TMX) involving O-methylene hydro-xylation and nitro reduction in the *A. bisporus* compost and casing soil.

In our study, we found that the parent pesticide TMX can be metabolized to CLO and TMX-urea after TMX application in compost and casing soil. The possible metabolite route of TMX in compost and casing soil is shown in Figure 3. The conversion of TMX to CLO was dependent on methylene hydroxylation via the cleavage of the oxadiazine, as was also proved by Kevin [14]. Hydrolysis under alkaline conditions promoted the transformation of TMX into TMX-urea, where the C=N bond on the ylidene (nitro) amine hexatomic ring was attacked by the hydroxyl (OH) group, providing a strong electron-withdrawing property caused by the NO_2_ group. TMX in tomato plants and a cell suspension culture could be metabolized into CLO, urea derivatives, and nitro guanidine and eventually be degraded into single heterocycle compounds [39]. Various studies reveal that the types and amounts of TMX metabolites might be related to protean factors in environmental conditions, matrix properties, application and analytical methods of pesticides, and so on [40].

**Figure 4:**
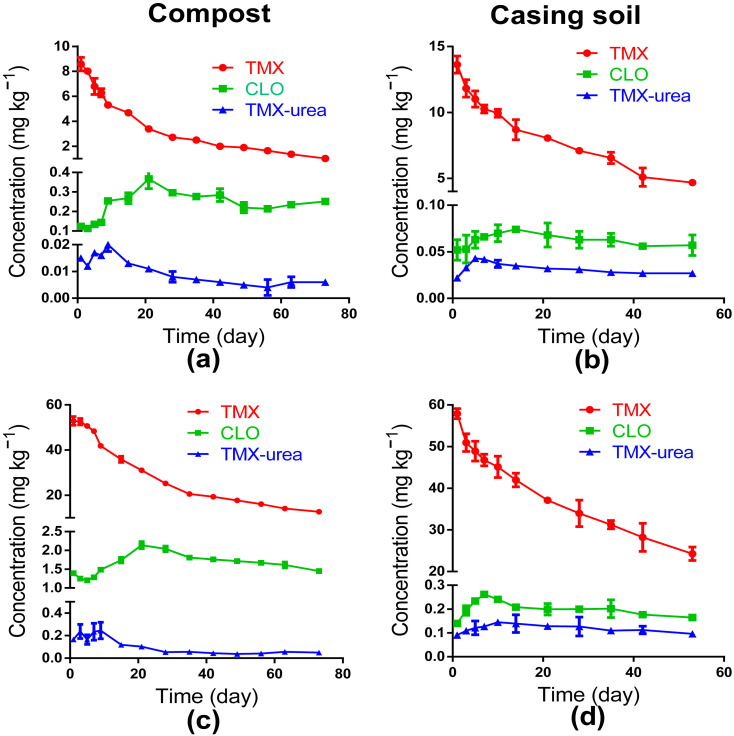
The concentration of parent pesticide thiamethoxam (TMX) and its two metabolites clothianidin (CLO) and thiamethoxam-urea (TMX-urea) during the dissipation dynamic of TMX applied in compost or casing soil at two dosage groups, respectively. (**a**) The 10 mg kg^−1^ application in compost; (**b**) the 10 mg kg^−1^ application in casing soil; (**c**) the 50 mg kg^−1^ application in compost; (**d**) the 50 mg kg^−1^ application in casing soil. Each treatment was performed in three replicate plots; data displayed are mean ± SD (*n* = 3).

The residual dynamics of parent TMX and its two metabolites, CLO and TMX-urea, when TMX is applied to compost or casing soil at two dosages are shown in Figure 4. CLO was found to be the dominant metabolite, no matter the dosage. In the casing soil of *A. bisporus*, the largest proportions of the two metabolites were 1.77% and 1.06% for dosages of 10 mg kg^−1^ and 50 mg kg^−1^ at 53 d after exposure, respectively. The results were similar to the previous research in JMPR 2010, where the total amount of most metabolites in soil was approximately 4–5% [41]. Meanwhile, in compost, the largest proportions of the two metabolites compared with parent TMX were 20.08% and 10.61% at the end point of 63 d after exposure to dosages of 10 mg kg^−1^ and 50 mg kg^−1^, respectively. The residual amounts of the two metabolites degraded from TMX in compost were much higher than in casing soil. The trends in their dissipation were similar in compost and casing soil. During the dissipation of TMX, the concentration of CLO increased steadily to the maximum levels, which were 0.367 mg kg^−1^ and 2.139 mg kg^−1^ for the low- and high-dosage groups, respectively, at 21 d in compost. Meanwhile, the maximum levels were 0.074 mg kg^−1^ at 14 d and 0.262 mg kg^−1^ at 7 d under 10 mg kg^−1^ and 50 mg kg^−1^ dosages in casing soil, and then decreased gradually until a balance was achieved. The residual dynamics of TMX-urea were the same as for CLO, first increasing to the highest concentration in 7–10 days, then slowly decreasing to equilibrium both in compost and in the casing soil for all dosage groups. The maximum concentration of TMX-urea in compost was 0.017 mg kg^−1^ at 5 d under a TMX dosage of 10 mg kg^−1^, and 0.245 mg kg^−1^ at 10 d under a dosage of 50 mg kg^−1^, respectively. Meanwhile, the maximum residue concentrations of TMX-urea in casing soil were 0.043 mg kg^−1^ at 5 d and 0.145 mg kg^−1^ at 10 d under the two dosage groups, respectively.

**Figure 5:**
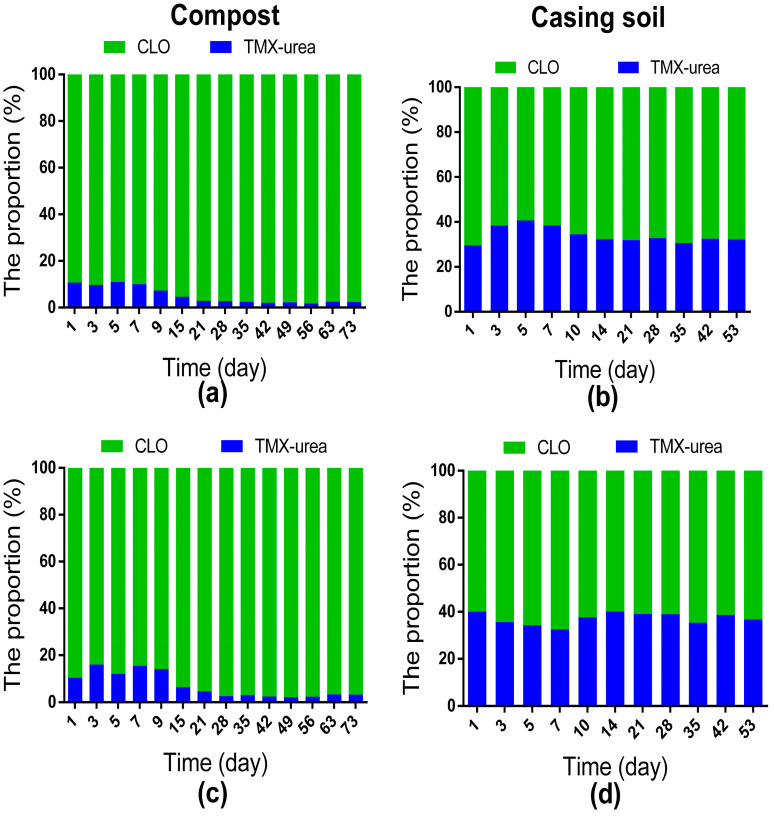
The proportion of clothianidin (CLO) and thiamethoxam-urea (TMX-urea) during the dissipation dynamic of TMX applied in compost or casing soil at two dosage groups, respectively. (**a**) the 10 mg kg^−1^ application in compost; (**b**) the 10 mg kg^−1^ application in casing soil; (**c**) the 50 mg kg^−1^ application in compost; (**d**) the 50 mg kg^−1^ application in casing soil. Each treatment was performed in three replicate plots; data displayed are mean values.

The proportions of CLO and TMX-urea in two metabolites during the dissipation dynamics of TMX applied in compost or casing soil at two dosages are shown in Figure 5. In the respective sampling period, the proportions of CLO remaining in the compost were obviously higher than in casing soil. The initial proportions of CLO in compost at 1 d were 89.21% and 89.37% under 10 mg kg^−1^ and 50 mg kg^−1^, respectively. Later, these proportions gradually increased to 98.17% at 56 d and 97.83% at 49 d for the 10 mg kg^−1^ and 50 mg kg^−1^ dosages, respectively. The overall proportions of TMX-urea in casing soil (29.73~40.57%) were significantly higher than in compost (1.83~15.95%). The detailed proportion trends of TMX-urea in casing soil were not exactly the same as in compost. The initial proportions of TMX-urea in casing soil at 1 d were 29.73% and 39.39% under 10 mg kg^−1^ and 50 mg kg^−1^, respectively. Later, these proportions increased to 40.57% at 5 d and 40.06% at 14 d, respectively, and the proportion decreased slightly in the later period until stable. On the other hand, the proportions of TMX-urea in compost rapidly rose to the highest points at 5 d with 11.26% and 3 d with 15.95% for dosages of 10 mg kg^−1^ and 50 mg kg^−1^, respectively, then dropped sharply.

### 3.4. Final Residues and Bioconcentration Factors (BCFs) in Fruiting Body

Residues and BCFs of TMX in fruiting bodies are summarized in Table 3. Neither TMX nor its two metabolites, CLO and TMX-urea, were detected in the fruiting bodies after TMX was applied to compost at two dosage groups. For TMX applied to casing soil, only parent compound TMX residues were detected, but no metabolites were found in fruiting bodies. Residual levels of TMX in the third flush mushrooms were lower than those in the first and second flush mushrooms. The results show that TMX applied in casing soil was able to migrate and accumulate in fruiting bodies, but not when applied to the compost. This may be related to the dissipation and metabolic behaviors of TMX in compost, as mentioned before. Based on these results, we inferred that TMX administered to compost was safer and more reasonable than TMX administered to casing soil. The BCFs of TMX from casing soil to fruiting bodies were between 0.0003 and 0.0006 at a 10 mg kg^−1^ dosage, while they were from 0.0007 to 0.0009 at a 50 mg kg^−1^ dosage. The values of BCFs were slightly higher in the high dosage group.

### 3.5. Dietary Risk Assessment

The risk assessment food category and consumption were downloaded from the ICAMA document [26]. The average capital mushroom intake, 0.0583 kg day^−1^, was obtained from the GEMS/Food consumption database [28]. The reference maximum residue limits (MRLs) used for the SMTRi of thiamethoxam were obtained from GB 2763-2021 and the EU-MRLs Database for Pesticides in Food [42,43]. The daily food intake and MRLs of thiamethoxam for urban and rural residents in China (Table S4) were selected to calculate the associated NEDI. The ADI and ARfD of TMX are 0.08 mg kg^−1^ bw day^−1^ and 1 mg kg^−1^ bw day^−1^, respectively, according to the JMPR report [41]. The STMR and HR residues of TMX in *A. bisporus* were 0.017–0.030 mg kg^−1^ and 0.019–0.033 mg kg^−1^, respectively, which were far below the MRL in GB 2763-2021, the value of which is 0.5 mg kg^−1^. The residual levels of TMX in fruiting bodies were positively correlated with dosage, which implies that the risk quotients of low dosages were lower than those of high dosages. As listed in Table 4, the RQ values of TMX in the first to third flush mushrooms were 0.1062~0.1064 when TMX was applied in the casing soil at a high dosage. RQ < 1 suggests that the chronic risk of TMX application in casing soil at 50 mg kg^−1^ was acceptable. The acute dietary risk assessments of TMX residue in *A. bisporus* fruiting bodies at a high dosage are listed in Table 5. The HQ values were in the range from 0.000018 to 0.000031, much less than 1. The results revealed that the estimated exposure levels were much lower than ARfD, which were considered too low to cause any toxicity, and so the exposure risks were negligible. All dietary risks are much lower than acceptable levels, which means that TMX application with ≤ 50 mg kg^−1^ in casing soil or compost will not pose dietary safety risks in *A. bisporus*.

## 4. Conclusions

In this study, an effective, improved QuEChERS coupled with the UPLC-MS/MS method was established and validated to simultaneously analyze TMX and its two metabolites, CLO and TMX-urea, in an *A. bisporus* cultivation ecosystem (casing soil, compost, and fruiting bodies). The dissipation and metabolism of TMX in casing soil or compost and the bioconcentration of TMX in *A. bisporus* cultivation were monitored and compared based on this method. The degradation rate of TMX in compost was evidently faster than that in casing soil. TMX can be metabolized to CLO and TMX-urea after TMX application in compost and casing soil; CLO was the predominant metabolite no matter the group. For TMX applied in casing soil, only parent compound TMX residues were detected, with residue levels ranging from 1.5 ± 0.3 μg kg^−1^ to 30.4 ± 2.2 μg kg^−1^ and BCFs between 0.0003 and 0.0009, but no metabolites were found in fruiting bodies. According to all the dietary risk assessments of TMX, NEDI and NESTI were far below the acceptable levels of ARfD and ADI, which means that the application of TMX at dosages under 50 mg kg^−1^ in casing soil or compost will not pose acute or chronic exposure risks to Chinese consumers. Overall, our study provides scientific evidence and offers a more comprehensive understanding of TMX uptake and metabolism by *A. bisporus* fruiting bodies, which will help in developing guidelines for the safe and reasonable application of thiamethoxam in *A. bisporus* cultivation. However, the effective dosage of TMX for pest control in *A. bisporus* cultivation still needs to be investigated. The more comprehensive and intensive metabolic mechanisms of TMX in compost and casing soil and the bioconcentration mechanism of TMX from casing soil to the fruiting body were insufficient. Therefore, more research will be required to apply TMX to pest control in *A. bisporus* cultivation.

## Figures and Tables

**Table 1:** Linear regression parameters of the calibration curve and LODs, LOQs, ME (%) of thiamethoxam and its metabolites in pure solvent and matrices.

Compound	Matrix	Calibration Range (mg L^−1^)	Regression Equation	*r*	LOD ^a^ (mg kg^−1^)	LOQ ^b^ (mg kg^−1^)	ME ^c^ (%)
Thiamethoxam	Acetonitrile	0.0001–1	*y* = 16,056.5*x* + 118,065.0	0.9916			
	Fruiting body	0.001–1	*y* = 19,785.9*x* + 26,133.7	0.9990	0.001	0.002	23.2
	Casing soil	0.005–1	*y* = 17,654.9*x* + 96,874.1	0.9915	0.005	0.01	9.9
	Compost	0.005–1	*y* = 18,068.9*x* + 24,581.9	0.9991	0.005	0.01	12.5
Clothianidin	Acetonitrile	0.0001–1	*y* = 12,036.1*x* + 50,954.7	0.9939			
	Fruiting body	0.001–1	*y* = 13,937.4*x* + 25,647.3	0.9980	0.001	0.002	15.8
	Casing soil	0.005–1	*y* = 13,713.6*x* + 26,238.5	0.9984	0.005	0.01	13.9
	Compost	0.005–1	*y* = 10,246.4*x* − 2445.0	0.9995	0.005	0.01	−14.9
Thiamethoxam-urea	Acetonitrile	0.0001–0.5	*y* = 78,973.9*x* + 31,132.0	0.9958			
	Fruiting body	0.0005–0.5	*y* = 81,351.6*x* − 4751.4	0.9915	0.0005	0.001	3.0
	Casing soil	0.001–0.5	*y* = 89,419.6*x* + 18,706.2	0.9927	0.001	0.002	13.2
	Compost	0.001–0.5	*y* = 61,454.2*x* + 19,152.3	0.9930	0.001	0.002	−22.2

^a^ Limit of detection; ^b^ Limit of quantification; ^c^ Matrix effect.

**Table 2:** The kinetic equations and half-lives of thiamethoxam in compost and casing soil at two dosage groups.

Matrix	Dosage (mg kg^−1^)	Kinetic Equation	R^2^	*t_1_*_/*2*_(d)
Compost	10	*C_t_* = 8.225*e*^−0.0351*t*^	0.9629	19.74
	50	*C_t_* = 54.32*e*^−0.024*t*^	0.9764	28.87
Casing soil	10	*C_t_* = 12.53*e*^−0.0207*t*^	0.9401	33.54
	50	*C_t_* = 53.98*e*^−0.0162*t*^	0.9438	42.89

**Table 3:** Bioconcentration factors (BCFs) and final residues of TMX in *A. bisporus* fruiting body after application in casing soil.

Matrix	Dosage (mg kg^−1^)	Compound	Final Residue (μg kg^−1^) (Days after Thiamethoxam Application)					
			First Flush *	BCF	Second Flush *	BCF	Third Flush *	BCF
Casing soil	10	thiamethoxam	4.3 ± 0.4	0.0006	2.6 ± 0.3	0.0005	1.5 ± 0.3	0.0003
	50	thiamethoxam	30.4 ± 2.2	0.0009	26.3 ± 2.4	0.0009	17.4 ± 1.9	0.0007

* The fruiting bodies were harvested at 28 d, 42 d, and 53 d after TMX application in casing soil, respectively; data displayed are mean ± SD (*n* = 5).

**Table 4:** The chronic dietary risk assessment of TMX residue in *A. bisporus* at high dosage group.

Matrix	ADI ^a^ (mg kg^−1^ *bw* day^−1^)	First Flush		Second Flush		Third Flush	
		Median Residue (mg kg^−1^)	RQ ^b^	Median Residue (mg kg^−1^)	RQ	Median Residue (mg kg^−1^)	RQ
Casing soil	0.08	0.03	0.1064	0.026	0.1063	0.017	0.1062

^a^ acceptable dietary intake; ^b^ chronic risk quotient.

**Table 5:** The acute dietary risk assessment of TMX residue in *A. bisporus* at high dosage group.

Matrix	ARfD ^a^ (mg kg^−1^ *bw* day^−1^)	First Flush		Second Flush		Third Flush	
		HR ^b^ (mg kg^−1^)	HQ ^c^	HR (mg kg^−1^)	HQ	HR (mg kg^−1^)	HQ
Casing soil	1	0.033	0.000031	0.029	0.000027	0.019	0.000018

^a^ acute reference dosage; ^b^ highest residue level; ^c^ acute risk quotient.

## Data Availability

Not applicable.

## Supplementary Materials

The following are available online at: https://www.mdpi.com/article/10.3390/toxics11060500/s1. Table S1: Composition and physicochemical properties of casing soil and compost. Figure S1: Diagrammatic representation of the growing stage of *Agaricus bisporus* production, day of pesticide application, and sampling. Table S2: Mass transition parameters of thiamethoxam, clothianidin, and thiamethoxam-urea for multiple reaction monitoring (MRM) using UPLC-MS/MS. Table S3: Recoveries and relative standard deviations (RSDs) of thiamethoxam and its metabolites in the fruiting body, casing soil, and compost at different spiked levels (*n* = 6). Table S4: The daily food intake and reference residue limits (MRLs) of thiamethoxam for urban and rural residents in China.
