# Supplementary material for: Dissipation, Bioconcentration and Dietary Risk Assessment of Thiamethoxam and Its Metabolites in Agaricus bisporus and Substrates under Different Application Methods
Source: Toxics. 2023 Jun 1;11(6):500. doi: 10.3390/toxics11060500 (PMC10305595; doi:10.3390/toxics11060500)
Supplement: Supplementary file 1 [file toxics-11-00500-s001.zip › toxics-2407656-supplementary.pdf]

Dissipation, Bioconcentration and Dietary Risk Assessment of Thiamethoxam and its Metabolites in *Agaricus bisporus* and Substrates Under Different Application Methods

Shanshan Chen [1,†], Qicai Zhang [1,†], Qinxiong Rao [1], Xianli Wang [1], Penghui Du [2] and Weiguo Song [1,*]

S1. Field trials with TMX application during *A. bisporus* cultivation

S1.1. A.bisporus cultivation

The procedures for industry mushroom cultivation including five phases: initial fermentation, secondary fermentation, inoculation and spawn run, casing soil and pre-cropping, cropping and harvesting. The compost (pH 6.5±0.2) was consisted of 52% wheat straw, 40.5% chicken manure, 3.5% Plaster, 2.4% cottonseed cake and 1.6% cottonseed husk (Table S1). They were mixed uniformity and then pile up a stack outdoors, turned at 3-4 day intervals for initial fermentation naturally. Transfer compost to a strict environmental controlled room for secondary fermentation, pasteurization process and cool down to 24-26 °C at the end, the phase II lasted 4-5 days. Sylvan A15 of *A. bisporus*, was inoculated as spawn mixed with the compost to complete inoculation subsequently. When the compost has been colonised with the mycelium, the casing soil (pH 7.8±0.2) composed of 80% peat soil, 17% water, 2.5% $CaCO_3$, 0.3% CaO, and 0.2% plaster (Table S1) should be covered. The highness of the casing soil covering the compost surface was 5-6 cm. The three flushes of fruiting bodies were harvested at 28 d, 42 d and 53 d after soil-casing, respectively. After the harvesting was completed, the whole cultivation period of *A. bisporus* was over.

Table S1. Composition and physicochemical properties of casing soil and compost.

Substrate	Composition (%)		MC[a] (%)	pH	OM[b] (%)	Ash (%)
Casing soil	Peat soil	80	69.2±0.4	7.8±0.2	35.9±1.3	57.2±1.8
	Water	17				
	$CaCO_3$	2.5				
	CaO	0.3				
	Plaster	0.2				
Compost	Wheat straw	52	62.1±0.5	6.5±0.2	65.9±1.6	33.6±1.2
	Chicken manure	40.5				
	Plaster	3.5				
	Cottonseed cake	2.4				
	Cottonseed husk	1.6				

[a] Moisture content; [b] Organic materials.

S1.2. TMX application and sampling

On the basis of the experimental design, for the group of compost treated with TMX (T1), 7.2 g and 36 g formulation of TMX (25% WDG) was dissolved sufficiently in water individually. Next, the both dosages of pesticide solutions were spraying respectively to 180

kg composts in a small stirring machine and spinning thoroughly to ensure the pesticides diffused uniformly. Per 15 kg composts with TMX were put in a basket (57 cm × 39 cm × 24 cm), each plot was comprised of four baskets and each treatment group had three replicate plots. In addition, the control group plot was performed the equal volume of water without TMX and equal amount of casing soil later without TMX. The equal number of baskets loaded composts without TMX were continued to cultivate till covering casing soil treated with TMX as the group of T2. The same dosage of casing soil was applied due to comparison of data analysis conveniently and scientifically. For TMX application in casing soil, 3.12 g and 15.6 g of 25 % TMX WDG was dissolved in water and separately applied to 78 kg casing soils and blending evenly with the stirring machine. The 6.5 kg TMX application casing soils were covered to each basket of T2. Approximate wet weight of 500 g compost and 500 g casing soil samples were collected randomly from each treatment plot at 2 h, 1, 3, 5, 7, 10, 14, 21, 28, 35, 42, 49, 56, 63, 73 d and 2 h, 1, 3, 5, 7, 10, 14, 21, 28, 35, 42, 53 d after TMX application, separately. The three flushes fruiting body were harvested at 28 d, 42 d and 53 d after covering casing soil. At these times, 1 kg of *A. bisporus* fruiting body samples were collected in each plot with the size 4-6 cm of the sporophore. Above all types of the samples from control plot were collected simultaneously at early, mid-term and the last harvesting period. Schematic diagram for arrangement of the pesticide application and sampling is outlined in Figure S1. The compost and casing soil samples were vacuum freeze-dried by Thermo HetoPower Dry LL1500 (Massachusetts, USA) and ground in a grinder to obtain the homogeneous powder, then put in a sealed plastic bag. The *A. bisporus* fruiting body samples were completely homogenized by a multi-function food processor (Oumaisi, Guangdong, China) and place into the plastic sample storage bottles. All samples were stored at -20 °C for further detection.

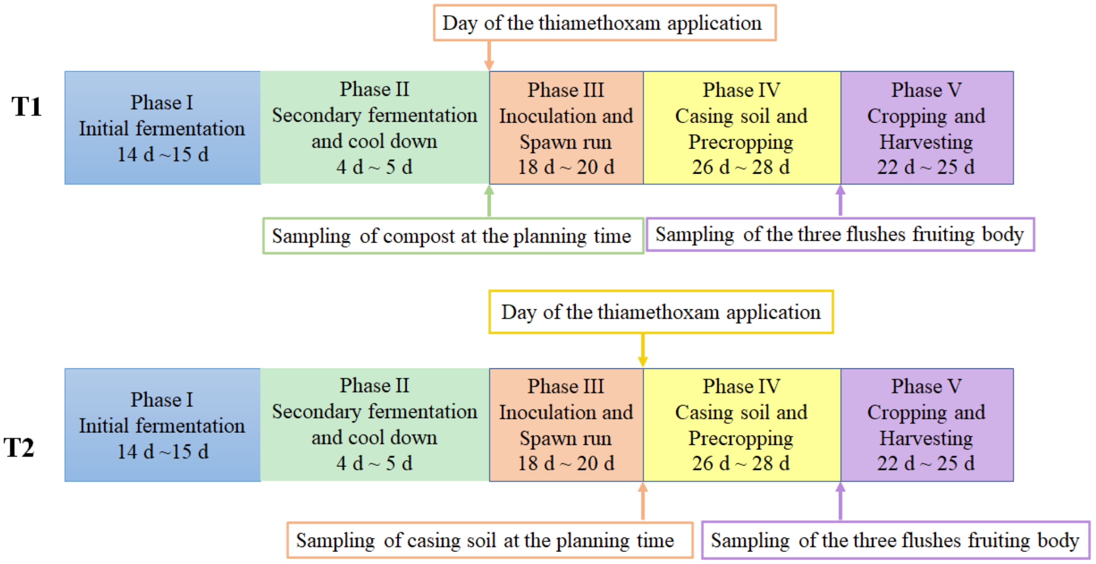

Figure S1. Diagrammatic representation of the growing stage of *Agaricus bisporus* production, day of the pesticide application and sampling.

T1: Compost treated with thiamethoxam;

T2: Casing soil treated with thiamethoxam;

Phase I: Mixing wetting straw , chicken manure and the other component into stack. Stack turned (mixed) at 3~4 day intervals. Temperature up to 75°C in the center of stack.

Phase II: Transfer compost to controlled environment room for pasteurization temperature of 55~63℃ for 3-4 h, cool down to 25℃at the end of period.

Phase III: Inoculation and colonization of substrate by mushroom mycelium.

Phase IV: Covering colonised substrate with casing soil, continuation of colonisation of compost and casing layer. Initiation and development of fruiting bodies at temperature on 16~20℃.

Phase V: Harvesting of the first flush up to three flushes. Flushes at intervals about 10-14 days. The three flushes fruiting body were harvested at 28 d, 42 d and 53 d after thiamethoxam application respectively.

Table S2. Mass transition parameters of thiamethoxam , clothianidin and thiamethoxam-urea for Multiple Reaction Monitoring (MRM) using UPLC-MS/MS.

Compound	Molecular formula	MW[a]	t_R[b] (min)	Precursor ion (m/z)	Product ion (m/z)	DP[c] (V)	CE[d] (eV)
Thiamethoxam	$C_8H_{10}ClN_5O_3S$	291.7	3.31	291.8$[M+H]^+$	211.0*,132.0	107,107	17,28
Clothianidin	$C_6H_8ClN_5O_2S$	249.7	3.76	250.0$[M+H]^+$	169.0*,132.0	55,80	19,26
Thiamethoxam-urea	$C_8H_{10}ClN_3O_2S$	247.1	3.99	248.1$[M+H]^+$	175.0*,132.0	108,88	28,27

[a] Molecular weight; [b] Retention time; [c] Declustering potential; [d] Collision energy.

*Quantification transition.

Table S3. Recoveries and relative standard deviations (RSDs) of thiamethoxam and its metabolites in fruiting body, casing soil and compost at different spiked levels (n=6).

Compound	Matrix	Spiked level (mg kg^{-1})	Average recovery (%)	RSD (%)
Thiamethoxam	Fruiting body	5	97.8	3.5
		1	97.1	4.3
		0.1	100.2	3.7
		0.02	94.5	4.6
		0.002	106.7	12.1
	Casing soil	5	97.7	7.1
		1	88.6	6.8
		0.1	89.1	6.3
		0.02	94.2	8.9
		0.01	113.4	6.7
	Compost	5	92.8	7.5
		1	90.4	8.1
		0.1	90.1	6.9
		0.02	91.2	9.2
		0.01	104.8	10.3
Clothianidin	Fruiting body	5	94.2	4.9
		1	92.9	6.1
		0.1	97.3	5.2
		0.02	92.6	8.9
		0.002	102.6	11.5
	Casing soil	5	86.1	6.3
		1	89.2	13.7
		0.1	90.0	4.8
		0.02	86.2	3.1
		0.01	94.8	12.4
	Compost	5	91.8	8.7
		1	90.9	9.1
		0.1	89.4	6.8

		0.02	88.2	5.9
		0.01	103.5	9.6
Thiamethoxam-urea	Fruiting body	5	90.1	4.8
		1	89.3	5.1
		0.1	101.2	4.3
		0.02	88.3	5.8
		0.001	95.6	8.4
	Casing soil	5	99.1	5.1
		1	86.3	4.2
		0.1	83.8	2.8
		0.02	85.6	4.6
		0.002	80.8	13.2
	Compost	5	89.7	6.4
		1	87.7	5.2
		0.1	91.7	7.0
		0.02	85.8	7.3
		0.002	89.3	14.1

Table S4. The daily food intake and reference residue limits (MRLs) of thiamethoxam for urban and rural residents in China.

Food classification	F_i (kg day^{-1})	Reference maximum residue limits (MRLs) or residue median values (mg kg^{-1})	Sources
Rice and its products	0.2399	0.1	China
Flour and its products	0.1385	0.1	China
Other cereals	0.0233	0.05	China
Tubers	0.0495	0.2	China
Dried beans and their products	0.016	0.04	China
Dark vegetables	0.0915	3	China
Light-colored vegetable	0.1837	1	China
Mushrooms	0.0583	-	This study
Pickles	0.0103	0.08	China
Fruits	0.0457	0.5	China
Nuts	0.0039	0.01	EU
Livestock and poultry	0.0795	0.01	China
Milk and its products	0.0263	0.05	China
Egg and its products	0.0236	0.01	China
Fish and shrimp	0.0301	-	-
Vegetable oil	0.0327	0.02	EU
Animal oil	0.0087	-	-
Sugar, starch	0.0044	0.01	EU
Salt	0.012	-	-
Soy sauce	0.009	-	-
Total	1.0869		

The risk assessment food category and consumption were downloaded from ICAMA, Institute for the Control Agrochemicals, Ministry of Agriculture and Rural Affairs of the People's Republic of China. (data from China pesticide information network); The average mushroom consumption per capita was obtained from GEMS/Food consumption database. The cluster diets were based on similarities between the dietary patterns. China belonged to G09, and the mean consumption of the mushroom was 0.0583 kg day^{-1} [28] (Reference in text). MRLs are searched from GB 2763-2021 National Food Safety Standard Maximum Residue Limits of Pesticides in Food and EU-Maximum Residue Limits Database for Pesticides in Food. Symbol "-" represents no information was found.
